# A case for conserving common species

**DOI:** 10.1371/journal.pbio.2004261

**Published:** 2018-02-07

**Authors:** Emmanuel A. Frimpong

**Affiliations:** Department of Fish and Wildlife Conservation, Virginia Tech, Blacksburg, Virginia, United States of America

This Perspective is part of the C*onservation Stories from the Front Lines Collection*

I grew up in a country where fish were, first and foremost, food. At the age of nine or so, I started following my dad and uncle to nearby rivers to fish on weekends, and soon I learned to make my own tackle and go back to the rivers to fish with my friends. We never caught a lot of fish, but every fish we caught came home with us to be eaten. It never occurred to us to consider what ecological role these fish might play or how they fit into the ecosystem. In Ghana, West Africa, like in almost all the developing countries in sub-Saharan Africa, viewing fish primarily as food flows naturally from the need to survive. Once survival is secured, humans can start thinking about other things, like prestige or social status, for example [1]. As I entered my postdoc in ecology and conservation—me, a Ghanaian who identified more with fisheries and natural resource management—I felt the distinction keenly. I had not studied for any of my degrees under brand-name ecologists or conservation scientists, which I had come to realize was helpful in launching an academic career in our field. I understood then that professional pedigree really matters. What I know now is that this is not merely for the prestige and access such mentorship provides but for the intellectual shelter and sustenance I must provide as a principal investigator (PI). This is the story of building my niche as an ecosystem engineer. But it is also the story of recognizing the ecological role of species we take for granted—and why we should fund research on common species that may sustain the rare ones, some of which we have yet to discover.

As a new PI, I would need to carve out my own niche to attract funding while also ensuring that my research program was interesting and topical enough to attract topnotch graduate students who would be proud to take a chance on my uncertain name recognition to advance their own careers. This is no small feat. My choice of research program was also constrained by the fact that my institution already had all sorts of established fish biologists, who studied game species; large river, reservoir, and marine ecosystems; and threatened and endangered species of streams. Of course, my colleagues had become established in these areas because that is where nearly all the research funding was and still is going.

Driven by considerations of cost and convenience, I decided to go local. I recruited my first group of students to study the fishes of the wadeable tributaries of the upper and middle New River in Virginia and North Carolina in the United States. Because it is a rather depauperate system with almost no fish species listed as of conservation concern by state or federal agencies, we had no competition. But we didn’t have much idea of what we were looking to do either. I had no particular species of interest in mind but vaguely planned to characterize the fish communities and determine the relationship between community structure and land use and land cover in the watersheds.

By summer that year, my team couldn’t help but notice that our “unremarkable” streams shared something truly remarkable: large mounds of gravel and pebbles, like underwater hills, littered the streambeds, every few meters in some cases. A single mound easily contained 10,000 to 20,000 rocks and attracted hundreds of fish of different species darting about in vivid colors—bluish, pink, yellow, and red, small but dazzling. On closer examination, we could see there was always at least one relatively large male maintaining or augmenting the mound nest by collecting rocks in its mouth and dropping them on the nest. Frequently, a nest would appear suddenly in a new location overnight and grow to its full size in 3 to 4 days. These constructions are the nests of the bluehead chub, *Nocomis leptocephalus*, of the minnow family (Cyprinidae; Fig 1). The genus *Nocomis* has nine recognized species and subspecies that occur in streams and rivers throughout eastern North America. With their ubiquity in my streams and this wonderful behavior, I decided that the *Nocomis* species demanded the spotlight and made it the focus of my early research.

**Fig 1:**
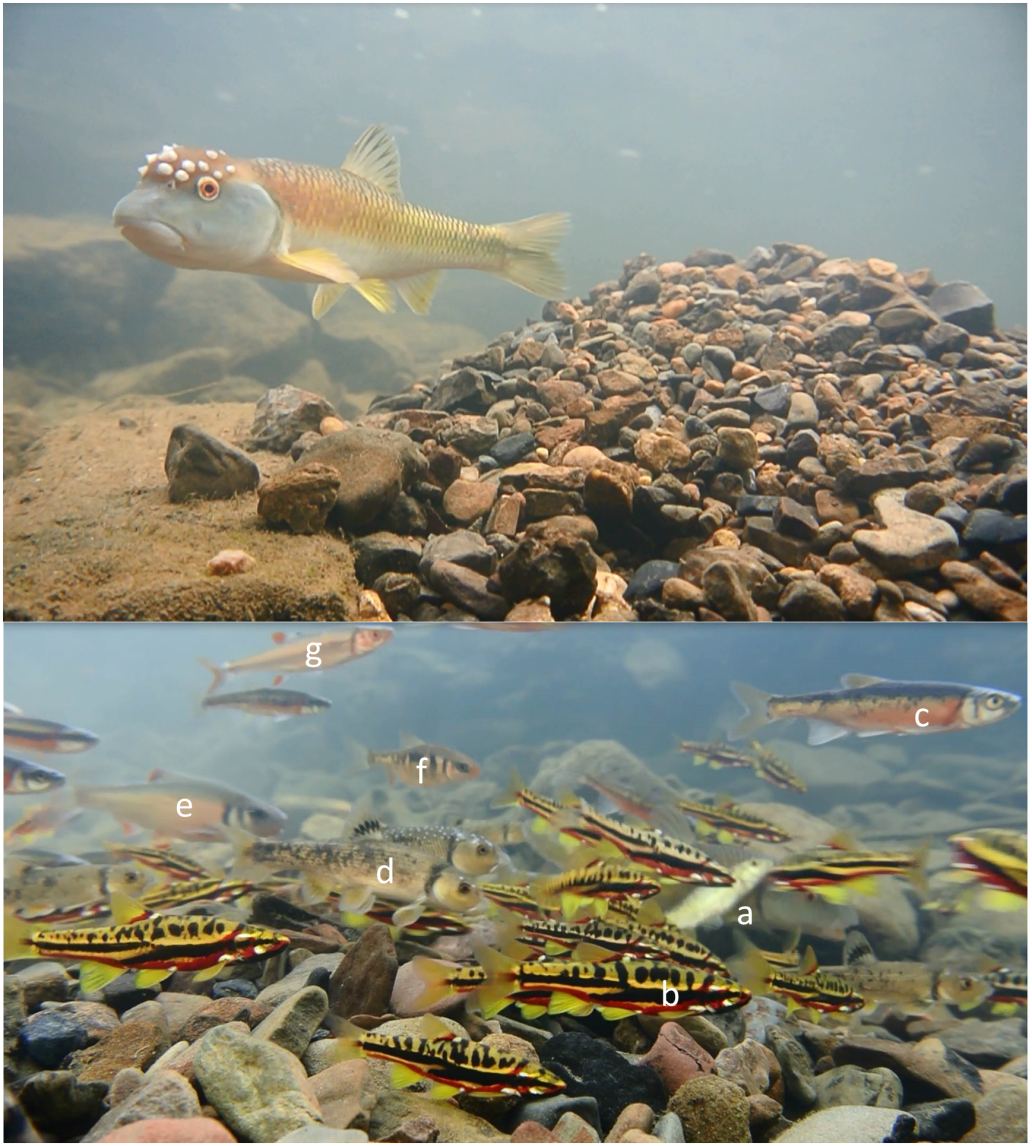
The nest and critical reproductive interactions around a common stream fish of North America. **Top**: A bluehead chub *N*. *leptocephalus* hovers over its completed and incubating mound nest. **Bottom**: An active nest with (a) host male bluehead chub initiating a spawning clasp and six nest associate species, including (b) mountain redbelly dace *Chrosomus oreas*, (c) rosyside dace *Clinostomus funduloides*, (d) central stoneroller *Campostoma anomalum*, (e) white shiner *Luxilus albeolus*, (f) crescent shiner *L*. *cerasinus*, and (g) rosefin shiner *Lythrurus ardens*. Photos are from Toms Creek, Virginia, US, by Emmanuel A. Frimpong.

I had so many questions: how does one 15-cm–long fish make a nest this large? Why does it need a nest this large? Why are all these other species using one species’ nest? Why does it let them? Which species are on the nest to spawn, and which species are there possibly with other motives? Why are there more nests in some streams than others? Why are *Nocomis* species so ubiquitous, and does their mode of reproduction have something to do with it? Natural historians had answered some of these questions [2,3], but the more conservation-oriented questions remained a mystery.

Over the course of 10 years, I would discover that *Nocomis* enjoys a mutualistic interaction with its nest associates in the New River ecosystem [4,5] and that the importance of this mutualism to the parties involved depends on the species, the threat of predation on spawning adults and their eggs, and abiotic factors [6]. I showed very early that nesting activity of the bluehead chub intensified in degraded habitats and that the nests would sometimes be the only useful habitat for the nest-associate species that required clean gravel and pebble substrate for spawning [7]. Minnows that depend strongly on *Nocomis* nests tend to have restricted ranges that are nested in the range of *Nocomis* species, thus explaining the geographic rarity of the obligate nest associates [8,9]. This also suggests that the relatively rare species owed their persistence in restricted ranges to *Nocomis* [7]. In June 2017, I observed nine different cyprinid species visit a *Nocomis* nest in one hour, and I have shown that at least seven of the nine species spawn on *Nocomis* nests sometime during the breeding season of about two months.

Nest association among freshwater fishes has now been reported across numerous families from nearly every continent, reinforcing the importance and pervasiveness of an ecological interaction that is woefully understudied and underreported. Even so, it’s clear that nest-building species that attract associates are keystone—if they are extirpated from an ecosystem, many dependent species will go with them (Table 1) [10]. Yet in the freshwater realm, no widely known aquatic conservation strategy is designed around our growing understanding of the importance of positive interdependence among species—biotic processes that may be just as important as physical habitat—not even in the regions with well-studied taxa such as the US. We think of species interactions as primarily negative (i.e., competitive, predatory, or parasitic) and default to prioritizing physical habitat protection or restoration of what is considered habitat for rare or declining species. We rush to save what’s visibly changing for the worse, be that a species, a habitat, or even a process. Therefore, although they are largely unrecognized, conservation of mutualisms has become a vital conversation for sustaining systems such as plant-pollinator complexes. But in doing species-by-species conservation, we lose sight of the fact that many rare species rely on positive interactions with common species. Can we protect these rare species without protecting the common species that function as their hosts? And what about species and interactions that are less visible or not yet fully understood?

**Table 1:** A representative community sample (collected with backpack electrofishing) from a 0.65-km section of Toms Creek, Virginia, showing dominance of the community by bluehead chub and its nest associates. Other fish species represented in significant numbers are those that feed actively on bluehead chub nests.

Species	Family	Number in Sample	Proportion of Community	Mode(s) of Reproduction	Nest Host	Trophic Link to Active *Nocomis* Nest
bluehead chub *Nocomis leptocephalus*	Cyprinidae	126	17.3	Mound nester	Host	No
rosyside dace *Clinostomus funduloides*	Cyprinidae	183	25.2	Nest associate	*Nocomis; Semotilus; Campostoma*	No
mountain redbelly dace *Chrosomus oreas*	Cyprinidae	99	13.6	Nest associate	*Nocomis; Semotilus; Campostoma*	No
central stoneroller *Campostoma anomalum*	Cyprinidae	57	7.8	Pit nester; Nest associate	*Nocomis; Semotilus*	Likely
creek chub *Semotilus atromaculatus*	Cyprinidae	29	4.0	Pit-ridge nester	-	Yes
white shiner *Luxilus albeolus*	Cyprinidae	20	2.8	Nest associate	*Nocomis*	No
blacknose dace *Rhinichthys atratulus*	Cyprinidae	16	2.2	Burry eggs in gravel and sand	-	Yes
rosefin shiner *Lythrurus ardens*	Cyprinidae	11	1.5	Nest associate	*Nocomis*	No
crescent shiner *Luxilus cerasinus*	Cyprinidae	10	1.4	Nest associate	*Nocomis*	No
rosyface shiner *Notropis rubellus*	Cyprinidae	3	0.4	Burry eggs in gravel and sand	-	No
New River shiner Notropis scabriceps	Cyprinidae	2	0.3	Broadcast spawn on rocks	-	No
telescope shiner *Notropis telescopus*	Cyprinidae	1	0.1	Broadcast spawn on rocks	-	No
white sucker *Catostomus commersonii*	Catostomidae	26	3.6	Burry eggs in gravel and sand	-	Yes
northern hogsucker *Hypentelium nigricans*	Catostomidae	17	2.3	Burry eggs in gravel and sand	-	Yes
blacktip jumprock *Moxostoma cervinum*	Catostomidae	3	0.4	Broadcast spawn on rocks	-	Yes
rock bass *Ambloplites rupestris*	Centrarchidae	15	2.1	Saucer-pit nest	-	Yes
redbreast sunfish *Lepomis auritus*	Centrarchidae	5	0.7	Saucer-pit nest	-	Yes
mottled sculpin *Cottus bairdii*	Cottidae	48	6.6	Crevice nest	-	Yes
margined madtom *Noturus insignis*	Ictaluridae	6	0.8	Crevice or burrow nest	-	Unknown
fantail darter *Etheostoma flabellare*	Percidae	50	6.9	Crevice nest	-	Yes

I return to my roots in grappling with this problem of how a lack of knowledge perpetuates ineffective practices. My introduction to fish ecology was Afrotropical freshwater fishes. Whenever I pick up a tuberculate male *Nocomis* or any of its associates these days, I can’t help but wonder what we’ve been missing under the turbid waters of tropical Africa. Memories of handling small tuberculate fishes in Ghana keep coming back from my junior fishing days. Now that I mentor students studying fish ecology in these two very different continents, I know that studying fish ecology and conservation in Africa can’t be all about fish farming and food security; there has to be a connection between my niches in the US and Africa. Perhaps it is just one splintered niche!

If fish are historically the most underfunded and understudied vertebrate group in conservation science [11], Afrotropical freshwater fishes are probably in the worst shape of them all. They may be the perfect example of where lack of knowledge perpetuates ineffectual conservation practices. Biodiversity in West and Central Africa is grossly under-sampled and the aquatic fauna mostly unstudied beyond occasional phylogenetic and taxonomic revisions. The regions are home to 600 to 800 described fish species [12], which are not only taxonomically unique as a group but perhaps represent an even larger global share of fish diversity than we currently assume. Globally, scientists have reported approximately one new species of fish per day for the past several decades [13]; and because fish are relatively invisible in their natural habitat, the number of fish species described from any ecosystem is essentially a function of the degree to which the system has been explored. The morphological variations in fishes I have observed while sampling in Ghana suggests that there are many undocumented, undescribed, and cryptic clusters of species lumped into one species in the West and Central Africa regions. The fishes of this region are basically “without status”—no real World Conservation Union (IUCN) Red List assessment has ever been conducted for many of them, or they are considered of no conservation concern on the basis of large range size or perceived local abundance from commercial and artisanal harvests. Unfortunately, rarity has many dimensions, and to the extent that rarity determines conservation status, a species with large range could still be imperiled considering its local population sizes or critical habitat specificity [9].

Meanwhile, West and Central Africa are teaming with dams, alluvial gold mining, petroleum oil extraction, urbanization, agriculture, and exotic fish introductions for aquaculture and a quietly growing aquarium interest. Many of the rarest species of West and Central Africa are listed under IUCN endangered or threatened status, with some ongoing habitat destruction and, occasionally, overexploitation for human consumption cited as the primary cause for concern. The most common species are nearly unanimously declared of no conservation concern. What if some of these common species sustain the rare species in various parts of their range through crucial but unknown facilitative biotic interactions?

Attempts at aquatic conservation in Africa largely mimic the approaches used in developed countries. However, to my knowledge, very little to no habitat monitoring and recovery efforts exist in West and Central African countries even for species that are listed under IUCN vulnerable categories. Setting aside or protecting land (watersheds) for the conservation of one rare and endangered species—often not the most prominent or economically valuable—is not easily supported even by people in developed countries due to perceived inconvenience and economic loss from disallowed activities. This approach also concentrates (often very limited) conservation resources on just a few species while, arguably, sometimes ignoring significant biological causes of species rarity and declines. Common species and the communities they inhabit may provide a better framework for conservation in Africa due to their prominence, economic importance usually as food, and potential unrecognized contributions to ecosystem functioning and sustaining rarer species.

The concept of conserving common species is not unheard of. The US Geological Survey Gap Analysis Program (GAP), for example, has a mission of “keeping common species common.” But even here, the practical conservation approaches are built around representation of habitats and species in managed and protected areas, not on any known biotic interactions among species. In fact, rare—and by implication, threatened and endangered—species are considered under the purview of a different government agency, the US Fish and Wildlife Service. But the GAP approach is an important first step that is adaptable to conserving species interaction networks involving both common and rare species, both in the US and Africa and elsewhere. Especially for Africa and its meager conservation resources, we need to rethink the often lamented, disproportionate allocation of resources to studying and conserving a few species while waiting for common species to become rare before we move in like heroes to manage a “crisis.”

Using this more inclusive framework, many common African freshwater fish species, just like the *Nocomis* species of eastern North America, would qualify as an umbrella, flagship, or keystone species or perhaps a combination of all three. These concepts have well-debated foundations in conservation biology [14]. The challenge will be to convince people back home that conserving fish biodiversity is just as much a part of the survival equation as eating fish and to convince conservation biologists that conserving common species offers a protective umbrella for rare species and interactions we have yet to discover. On the one hand, I see a naturally easy case to make to a society that values its fish as food to conserve the habitat and interspecific interactions for economically valued and widespread species. However, I have to admit that conservation biologists must first face the daunting task of understanding the ecology and natural history of individual species in order to target species and processes for conservation with maximum benefit.

But I believe there’s a way to combine both challenges. Students of aquatic sciences in Africa need to be taught basic ecology and conservation, not just how to create ever more food out of fish. I am convinced that many novel biotic interactions await discovery in Afrotropical freshwater ecosystems and that a research agenda that is increasingly focused on understanding the ecology and natural history of Afrotropical fishes will deliver rich rewards, not just to students but to the ecosystem as well. Perhaps I didn’t need to travel to another continent to see the novelty of nature. But doing so has shown me that providing shelter and sustenance to others—in streambeds or academia—can have unforeseen and far-reaching benefits. Although there are many scientists who have spent whole careers studying Afrotropical fishes, especially their taxonomy and phylogeny, so much remains unknown. I am calling humbly on all active fish biologists, African governments, the IUCN, international aid agencies of developed countries, and foundations that sponsor research in ecology and nature conservation in Africa to respond to the dearth of knowledge of Afrotropical fishes as we would any biodiversity crisis!

## Abbreviations

GAP: Gap Analysis Program
IUCN: World Conservation Union
PI: principal investigator
